# Occupational class and risk of renal cell cancer

**DOI:** 10.1002/hsr2.49

**Published:** 2018-05-16

**Authors:** Masayoshi Zaitsu, Adolfo G. Cuevas, Claudia Trudel‐Fitzgerald, Takumi Takeuchi, Yasuki Kobayashi, Ichiro Kawachi

**Affiliations:** ^1^ Department of Social and Behavioral Sciences Harvard T.H. Chan School of Public Health Boston Massachusetts USA; ^2^ Department of Public Health, Graduate School of Medicine The University of Tokyo Tokyo Japan; ^3^ Department of Community Health Tufts University Medford Massachusetts USA; ^4^ Department of Urology Kanto Rosai Hospital Kawasaki Kanagawa Japan

**Keywords:** hypertension, job stress, occupational class, renal cell cancer, smoking

## Abstract

**Objectives:**

We sought to examine the association between occupational class linked to job stress and the risk of renal cell cancer. To identify potential mediators, we additionally examined whether any observed associations persisted even after controlling for the contribution of stress‐related factors (eg, smoking, hypertension, and obesity).

**Methods:**

Using nationwide inpatient records (1984 to 2016) from the Rosai Hospital group in Japan, we identified 3316 cases of renal cell cancer (excluding upper tract urothelial cancer) and 168 418 controls. We classified patients' occupational class (blue‐collar workers, service workers, professionals, and managers) and cross‐classified it by industry type (blue‐collar, service, and white‐collar) based on a standardized national classification. Unconditional logistic regression with multiple imputation was used for the analyses.

**Results:**

A significantly elevated risk of renal cell cancer was found among men in higher occupational class (eg, professionals and managers). The elevated odds in male managers across all industries persisted even after controlling for smoking and alcohol consumption, with the association being more pronounced in blue‐collar industries (OR, 1.61; 95% CI, 1.34‐1.93). The association appeared to be mainly mediated by hypertension.

**Conclusion:**

Occupational class is associated with the risk of renal cell cancer in men, particularly through modifiable risk factors.

## 1 INTRODUCTION

Renal cell cancer accounts for 2% of all malignancies in Japan, and the incidence has been increasing in recent years.^1^, ^2^, ^3^ In 2013, Cancer Information Service, National Cancer Center, Japan, estimated that the total incidence of kidney cancer (including upper tract urothelial cancer) was 24 865 (16 610 male and 8 255 female).^4^ Growing evidence suggests that stress‐related risk factors—eg, smoking, obesity, and hypertension^5^, ^6^, ^7^—contribute to the risk of renal cell cancer.^8^, ^9^, ^10^, ^11^, ^12^, ^13^, ^14^ However, very little is known of the role that stress plays in the risk of renal cell cancer, and the association between hypertension and the risk of renal cell cancer has been previously undocumented in Japan.

Stress has long been hypothesized as a possible contributor to cancer risk via stress coping responses (ie, an increase in coping behaviors such as smoking or excess drinking), and/or direct physiological responses (eg, elevated blood pressure) that is partially mediated by activation of the sympathetic nervous system, inflammatory pathways, and the hypothalamic‐pituitary‐adrenal axis.^15^, ^16^ However, the empirical evidence linking various dimensions of stress to cancer incidence has remained inconsistent.^17^, ^18^ Regarding work‐related stress, in the Nurses' Health Study, there was no association between multiple aspects of job stress, such as high demands and low control as well as low social support at work, and breast cancer or ovarian cancer.^19^, ^20^ Similarly, meta‐analyses have not found an association between work stress and lung, colorectal, breast, or prostate cancer.^21^ Yet no study to date has specifically investigated the relationship between stress because of work characteristics and renal cell cancer risk.

In Japanese society, higher occupational classes (managers and professionals) tend to report more job stress,^22^, ^23^ particularly following the collapse of the “economic bubble” in 1990. For example, Suzuki et al found that the occupational gradient in suicide in Japan reversed during the last 30 years.^22^ Specifically, prior to the economic collapse of the asset bubble in 1991, suicide rates were higher among service, sales, and production workers. In the decades following the collapse, however, suicide rates have been higher among professional and managerial workers.

The distribution of job stress is markedly different in the Japanese workplace compared with the United States. For example, a recent study in Japan indicated that higher psychological distress in administrative and professional occupations is associated with increased cancer mortality at several sites.^24^ Another study showed that the age‐standardized suicide mortality rate increased among Japanese male administrative/managerial workers^22^ between 1975 and 2005. In the same study, the lowest odds for suicide was observed among blue‐collar production workers.^22^ More recently, Tanaka et al^25^ reported that the age‐adjusted mortality rate for male managers increased across 12 types of occupation during the period of 1995 to 2010, which straddles the global economic crisis of 2008. While the magnitude of job stress across occupational classes is debated,^26^, ^27^ higher occupational class does indeed appear to be related to greater job stress in Japanese society, as indicated by the higher rates of suicide rates among managers and professionals in Japan.^22^, ^23^ Hence, in contrast to US/European studies, which typically show that job stress is higher among low‐status occupations compared with high‐status ones, the opposite pattern is found in Japan. Additionally, the prevalence of both hypertension and unipolar depression appeared to be higher in white‐collar occupations compared with blue‐collar occupations in Japan,^28^, ^29^ and hypertension appeared to be linked to job stress.^28^

In the present study, we sought to examine the association between occupational class and renal cell cancer, assuming that occupational class is a proxy for work‐related stress.^30^, ^31^ In addition, we assumed that occupational class is associated with stress‐related factors (smoking, hypertension, and obesity), and that these may increase the risk for renal cell cancer. Therefore, we also tested whether any observed renal cell cancer risk associated with occupational class persisted even after controlling for the potential mediation by stress‐related factors.

## 2 MATERIALS AND METHODS

We conducted a hospital‐based case‐control study using inpatient electronic medical records of the Rosai Hospital group run by the Japan Organization of Occupational Health and Safety, an independent administrative agency. Details of the study database have been previously described.^32^, ^33^ Briefly, the Rosai Hospital group consists of 34 general hospitals in the main urban areas of Japan. Since 1984, the hospitals have recorded information on the clinical and occupational history of all inpatients. The database includes basic sociodemographic characteristics of patients, clinical diagnoses, and occupational history, as well as patients' smoking and alcohol habits, derived from questionnaires completed at the time of admission. Since 2002, pathological diagnoses have been recorded for cancer cases, while information on other risk factors (eg, hypertension, diabetes, and obesity) has been recorded since 2005. Trained registrars or nurses are responsible for registering the data. Occupational history is coded according to the standardized national classification (viz, the Japan Standard Occupational Classification and Japan Standard Industrial Classification) corresponding, respectively, to the International Standard Industrial Classification and International Standard Occupational Classification.^32^, ^33^ Written informed consent was obtained before patients completed the questionnaires.

We obtained a dataset under the research agreement between the authors and the Japan Organization of Occupational Health and Safety. The Research Ethics Committees of Graduate School of Medicine, The University of Tokyo, Tokyo (Protocol No. 3890‐3) and Kanto Rosai Hospital, Kanagawa, Japan (Protocol No. 2014‐38) approved the study.

### 2.1 Cases and controls

The study subjects comprised 171 734 patients (3316 cases of renal cell cancer [excluding upper tract urothelial cancer] and 168 418 hospital controls) aged 20 years or older, admitted to hospitals between April 1984 and March 2016. According to available national statistics estimated with several high‐quality local cancer registries in Japan, the total number of renal cell cancer cases in our data set represents 0.8% of the total incidence of kidney cancer (including upper tract urothelial cancer) in Japan for the years 1984 to 2013 (3033 of 357 993).^4^

We excluded patients with the diagnosis of upper tract urothelial cancer or patients with preexisting cancer history from the cases. Controls were patients diagnosed with musculoskeletal diseases (ICD‐9, 410‐739 and ICD‐10, M00‐M99; 89%) and skin diseases (ICD‐9, 680‐709 and ICD‐10, L00‐L99; 11%). We assumed that these diagnoses selected for the control groups were not linked to work stress.^34^

### 2.2 Occupational class defined by occupational and industrial category

The questionnaire included questions about the patients' current job and their 3 most recent ones (including age at starting and ending). The occupations were coded with 3‐digit codes in Japan Standard Occupational Classification for occupation category and 3‐digit codes in Japan Standard Industrial Classification for industry category. We selected the longest held job from the history for each patient.

**Figure 1:**
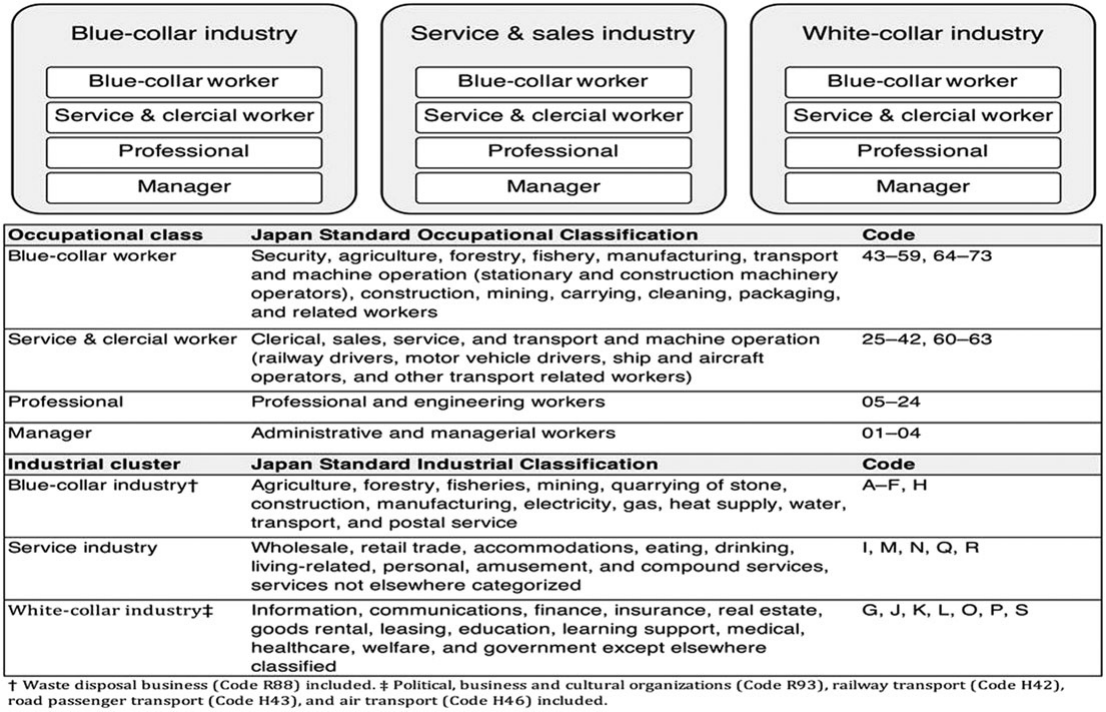
Occupational class cross‐classified with industrial cluster

Owing to the enormous variety of “longest held” jobs, we aggregated the occupations into 4 occupational classes, based on previous studies^26^, ^27^, ^35^, ^36^: “blue‐collar workers,” “service and clerical workers,” “professionals,” and “managers.” We also categorized the longest held occupations into 3 industrial clusters based on the methodology used in a previous study^37^: “blue‐collar industry,” “service and sales industry,” and “white‐collar industry” (Figure 1). We excluded those who were not actively engaged in paid employment (eg, homemakers, students, and unemployed) in the present study. In addition, we excluded female managers in the white‐collar industry because we did not observe any renal cell cancer cases in that category.

### 2.3 Covariates

Age and year of hospital admission were adjusted as confounding factors. To control potential changes in diagnosis and treatments over time, we adjusted for year of hospital admission. In mediation models, we included smoking and alcohol consumption, as well as potential stress‐related factors such as hypertension, obesity, and diabetes, as mediators. We assumed that occupational class is associated with stress‐related risk factors (smoking, hypertension, and obesity), and that these may increase the risk for renal cell cancer.

### 2.4 Statistical analysis

Among study subjects, 11% did not provide information on occupational history, smoking, and alcohol consumption and 20% did not complete all data. The background characteristics differed between those with complete and incomplete data (Table S1), and excluding incomplete data may lead to biased inference.^38^, ^39^ To deal with missing data, we performed multiple imputation for missing data among the 171 734 study subjects using all data, including occupational class, smoking, and alcohol consumption.^38^, ^39^, ^40^ Five imputed datasets were generated with multiple imputation by chained equations method^39^, ^40^; the following missing data were multiply imputed: occupational class (20 359, 12%), smoking (23 692, 14%), and alcohol consumption (48 608, 28%).

Using unconditional logistic regression with multiple imputation, we estimated the odds ratios (ORs) and 95% confidence intervals (CIs) for renal cell cancer in each occupational class, with blue‐collar workers in the blue‐collar industry as the reference group. We pooled the 5 ORs and 95% CIs obtained from each imputed dataset into one combined OR and 95% CI.^39^, ^40^ We stratified all the regression models by sex. First, we estimated the OR and 95% CI adjusted for age and year of hospital admission (model 1). Next, we adjusted for age, year of admission, and smoking (model 2). Finally, we additionally adjusted for drinking (model 3).

Owing to the data limitation that the other stress‐related factors (ie, hypertension, diabetes, and obesity) were only available after 2005, we evaluated the contribution of hypertension, diabetes, and obesity among 63 704 patients admitted to hospitals after 2005 (1544 cases and 62 160 controls). The following missing data were multiply imputed: occupational class (6943, 11%), smoking (6968, 11%), alcohol consumption (19 198, 30%), hypertension (8507, 13%), diabetes (8508, 13%), and obesity (8508, 13%). In subgroup analysis, we checked for a mediation by hypertension diagnosis (model 4). Finally, in model 5, we controlled for all covariates for hypertension, diabetes, and obesity, as well as age, year of hospital admission, smoking, and drinking.

Owing to the selection of hospital controls that might introduce selection bias in either direction (ie, toward or away from the null), we performed sensitivity analysis with 2 different alternative control groups: (1) all available controls diagnosed with all benign diseases (3316 cases and 1 298 207 controls) and (2) controls diagnosed with musculoskeletal disease (3316 cases and 150 210 controls). Additionally, we performed unconditional logistic regression among patients with complete data without performing multiple imputation (2496 cases and 116 139 controls diagnosed with musculoskeletal and skin diseases).

Alpha was set at 0.05, and all *P* values were 2‐sided. Data were analyzed using STATA/MP13.1 (Stata‐Corp LP, College Station, Texas).

## 3 RESULTS

**Figure 2:**
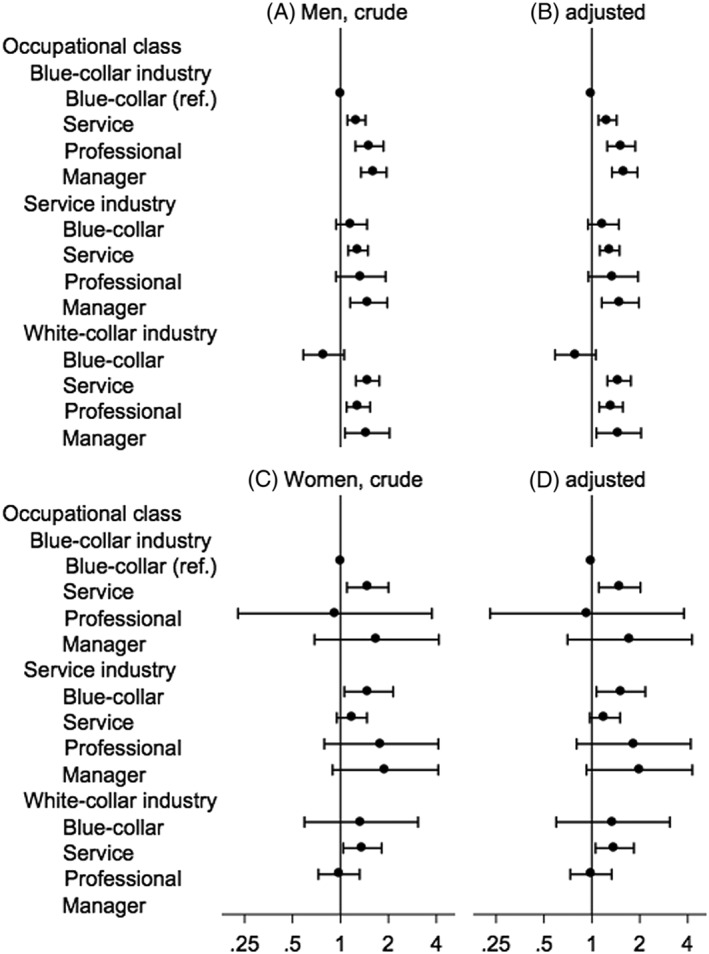
Odds ratios for renal cell cancer across different occupational classes stratified by sex. The odds ratio (dot) and 95% confidence interval (bar) were estimated by unconditional logistic regression with 5 imputed data. Male and female odds ratios were (A, C) adjusted for age and year of admission and (B, D) additionally adjusted for smoking and drinking. The numbers of cases and controls were, respectively, 2703 and 111 925 for men and 613 and 56 493 for women

Among men, those in higher occupational class (professionals and managers) had a significantly increased risk of renal cell cancer compared with blue‐collar workers across all industry types (Figure 2). In all 3 industries, men in the highest occupational groups, ie, managers, had significantly increased risk for renal cell cancer, with minimally adjusted OR ranging from 1.47 (for managers in the white‐collar industry) to 1.62 (for managers in the blue‐collar industry; Table 1). The observed increased OR for managers in all industries were not attenuated on adjustment for covariates and remained significantly associated with the risk for renal cell cancer on adjustment for covariates (adjusted OR ranged from 1.48 for managers in the white‐collar industry to 1.61 for managers in the blue‐collar industry, model 3; Table 1).

**Table 1:** Odds ratios in each occupational class associated with risk for renal cell cancer

Characteristics	Control, %^a^	Case, %^a^	Odds Ratio (95% Confidence Interval)^a^		
			Model 1^b^	Model 2^c^	Model 3^c^
Men					
Total number	111 925	2703			
Occupational class					
Blue‐collar industry					
Blue‐collar worker	39.0	34.2	1.00	1.00	1.00
Service worker	13.5	14.2	1.26 (1.11‐1.44)	1.26 (1.10‐1.43)	1.26 (1.10‐1.43)
Professional	4.3	5.0	1.52 (1.24‐1.86)	1.53 (1.25‐1.88)	1.53 (1.25‐1.87)
Manager	3.2	5.8	1.62 (1.35‐1.95)	1.61 (1.34‐1.94)	1.61 (1.34‐1.93)
Service industry					
Blue‐collar worker	4.7	4.0	1.17 (0.94‐1.47)	1.18 (0.94‐1.47)	1.18 (0.94‐1.48)
Service worker	13.4	13.2	1.29 (1.12‐1.49)	1.29 (1.12‐1.49)	1.29 (1.12‐1.49)
Professional	1.1	1.2	1.34 (0.94‐1.92)	1.36 (0.95‐1.95)	1.36 (0.95‐1.95)
Manager	1.6	2.7	1.50 (1.15‐1.97)	1.51 (1.15‐1.97)	1.51 (1.15‐1.97)
White‐collar industry					
Blue‐collar worker	3.6	2.0	0.78 (0.58‐1.05)	0.79 (0.59‐1.06)	0.79 (0.59‐1.06)
Service worker	8.1	9.6	1.48 (1.25‐1.75)	1.48 (1.25‐1.76)	1.48 (1.25‐1.76)
Professional	6.5	6.5	1.29 (1.09‐1.53)	1.32 (1.11‐1.56)	1.32 (1.11‐1.57)
Manager	1.0	1.7	1.47 (1.07‐2.03)	1.48 (1.07‐2.04)	1.48 (1.07‐2.04)
Age, mean (SD), y	50 (17)	62 (12)	1.05 (1.04‐1.05)	1.04 (1.04‐1.05)	1.05 (1.04‐1.05)
Year of admission, mean (SD)	2000 (8)	2003 (8)	1.02 (1.01‐1.03)	1.02 (1.01‐1.03)	1.02 (1.01‐1.03)
Smoking					
Never	27.0	25.4		1.00	1.00
≤20 pack‐year	30.3	19.9		0.93 (0.82‐1.06)	0.92 (0.81‐1.05)
>20‐40 pack‐year	25.7	29.6		1.15 (1.03‐1.28)	1.13 (1.01‐1.26)
>40 pack‐year	16.9	25.1		1.13 (1.01‐1.26)	1.10 (0.98‐1.24)
Daily alcohol intakes					
Never	24.7	23.8			1.00
≤15 g	6.7	6.0			0.98 (0.79‐1.20)
>15‐30 g	29.3	31.7			1.07 (0.96‐1.19)
>30 g	39.3	38.4			1.10 (0.96‐1.25)
Women					
Total number	56 493	613			
Occupational class					
Blue‐collar industry					
Blue‐collar worker	28.9	28.1	1.00	1.00	1.00
Service worker	8.8	10.0	1.48 (1.10‐2.00)	1.49 (1.10‐2.01)	1.49 (1.11‐2.02)
Professional	0.5	0.3	0.92 (0.23‐3.75)	0.92 (0.23‐3.76)	0.93 (0.23‐3.79)
Manager	0.5	0.8	1.69 (0.69‐4.15)	1.70 (0.69‐4.18)	1.73 (0.70‐4.25)
Service industry					
Blue‐collar worker	4.5	6.4	1.50 (1.06‐2.14)	1.52 (1.07‐2.16)	1.52 (1.07‐2.17)
Service worker	28.2	28.1	1.18 (0.95‐1.47)	1.20 (0.97‐1.50)	1.21 (0.97‐1.50)
Professional	0.8	1.0	1.81 (0.79‐4.12)	1.82 (0.80‐4.14)	1.83 (0.80‐4.18)
Manager	0.6	1.1	1.91 (0.89‐4.11)	1.97 (0.91‐4.23)	1.99 (0.92‐4.27)
White‐collar industry					
Blue‐collar worker	0.9	1.0	1.35 (0.59‐3.07)	1.35 (0.59‐3.08)	1.36 (0.60‐3.09)
Service worker	12.0	12.9	1.37 (1.04‐1.81)	1.38 (1.05‐1.82)	1.39 (1.05‐1.84)
Professional	14.5	10.4	0.98 (0.73‐1.32)	0.98 (0.73‐1.32)	0.99 (0.73‐1.33)
Manager	NA	NA	NA	NA	NA
Age, mean (SD), y	54 (17)	61 (13)	1.03 (1.02‐1.03)	1.02 (1.02‐1.03)	1.02 (1.02‐1.03)
Year of admission, mean (SD)	2001 (9)	2003 (8)	1.04 (1.02‐1.06)	1.04 (1.02‐1.06)	1.04 (1.02‐1.06)
Smoking					
Never	78.6	85.0		1.00	1.00
≤20 pack‐year	16.0	8.7		0.64 (0.47‐0.85)	0.65 (0.48‐0.88)
>20‐40 pack‐year	4.4	5.2		1.04 (0.72‐1.49)	1.06 (0.73‐1.54)
>40 pack‐year	1.0	1.1		0.86 (0.41‐1.83)	0.88 (0.41‐1.89)
Daily alcohol intakes					
Never	68.5	74.5			1.00
≤15 g	10.2	7.2			0.81 (0.55‐1.19)
>15‐30 g	16.1	14.3			0.98 (0.76‐1.26)
>30 g	5.2	3.9			0.89 (0.57‐1.40)

Abbreviation: NA, not available.

a Data were estimated with 5 imputed datasets. The percentage may not total 100 because of rounding and multiple imputation. The study period from April 1984 to March 2016 was divided into 2‐year financial years.

b Unconditional logistic regression with multiple imputation, adjusted for age and year of admission (confounders, model 1).

c Additional adjustment for smoking (mediators, model 2); smoking and alcohol consumption (mediators, model 3).

Among women, we observed marginal increases in the risks for managers (Figure 2). The results in the minimal‐adjusted and full‐adjusted models were similar (Table 1). The full‐adjusted risk of managers and professionals in the service and sales industry were marginally elevated (model 3; Table 1).

**Figure 3:**
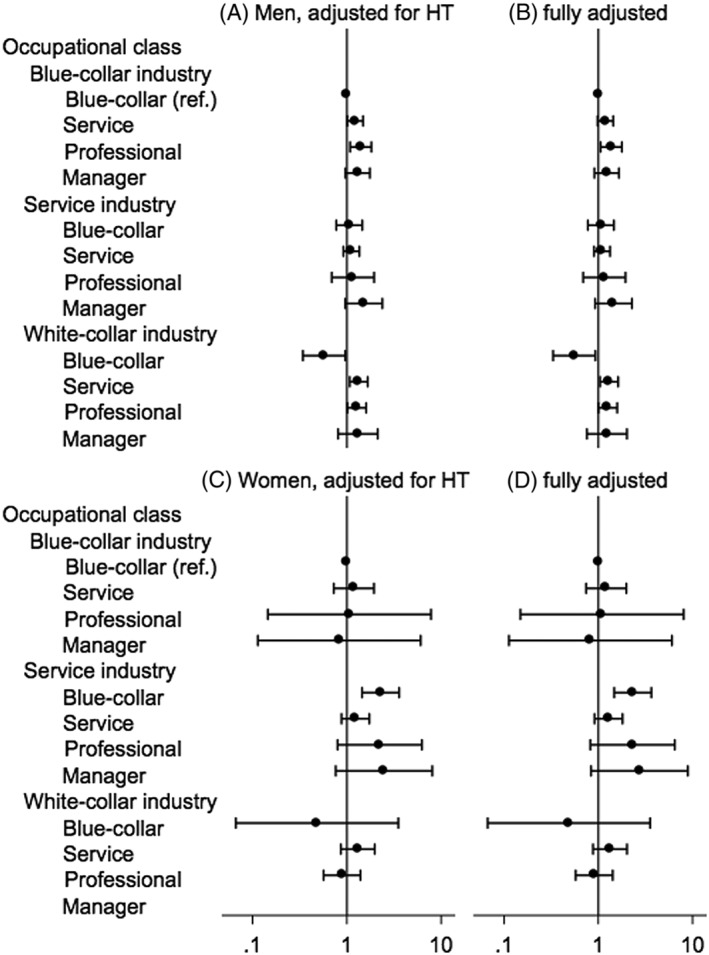
Odds ratios adjusted for hypertension and other stress‐related factors in a subset data after 2005. The odds ratio (dot) and 95% confidence interval (bar) were estimated by unconditional logistic regression with 5 imputed data. Male and female odds ratios were (A, C) adjusted for age, year of admission, and hypertension and (B, D) fully adjusted for hypertension, diabetes, obesity, age, year of admission, smoking, and drinking. The numbers of cases and controls were, respectively, 1265 and 41 097 for men and 279 and 21 063 for women

In the subgroup analysis, the gradient of the ORs across occupational classes showed the same trend (Figure 3). Among men, lifestyle‐related diseases (hypertension, diabetes, and obesity) were independently associated with the risk for renal cell cancer (eg, hypertension, OR 1.36; 95% CI, 1.20−1.54; model 5; Table 2); the elevated risk for higher occupational class was attenuated largely by adjustment for hypertension (model 4). After fully adjusting for all potential mediating factors, the risk for higher occupational class was not significant (except for professionals in blue‐collar and white‐collar industries; model 5). Among women, the fully adjusted risk among higher occupational class workers was not significantly elevated (Figure 3); however, the odds in the service and sales industries showed a trend suggesting a positive occupational gradient pattern (ie, higher risk with higher occupational class; model 5; Table 2).

**Table 2:** Subgroup analysis for mediation with hypertension and other stress‐related factors after 2005

Characteristics	Control, %^a^	Case, %^a^	Odds Ratio and 95% Confidence Interval^a^	
			Model 4^b^	Model 5^c^
Men				
Total number	41 097	1265		
Occupational class				
Blue‐collar industry				
Blue‐collar worker	35.3	32.3	1.00	1.00
Service worker	14.1	15.7	1.22 (1.01‐1.47)	1.19 (0.98‐1.44)
Professional	5.0	5.9	1.39 (1.08‐1.81)	1.37 (1.06‐1.78)
Managers	3.0	4.4	1.28 (0.95‐1.73)	1.23 (0.91‐1.66)
Service industry				
Blue‐collar worker	4.9	4.2	1.07 (0.78‐1.47)	1.07 (0.77‐1.46)
Service worker	14.0	13.0	1.11 (0.91‐1.35)	1.10 (0.90‐1.33)
Professional	1.2	1.3	1.17 (0.70‐1.95)	1.16 (0.69‐1.94)
Managers	1.5	2.6	1.49 (0.94‐2.34)	1.45 (0.93‐2.28)
White‐collar industry				
Blue‐collar worker	3.8	1.7	0.57 (0.34‐0.96)	0.56 (0.33‐0.93)
Service worker	8.6	9.9	1.32 (1.06‐1.65)	1.30 (1.04‐1.62)
Professional	7.5	7.7	1.27 (1.01‐1.60)	1.26 (1.00‐1.59)
Manager	1.1	1.5	1.28 (0.79‐2.08)	1.24 (0.76‐2.01)
Age, mean (SD), y	55 (17)	63 (12)	1.03 (1.03‐1.04)	1.03 (1.03‐1.04)
Year of admission, mean (SD)	2010 (3)	2010 (3)	1.05 (1.01‐1.09)	1.05 (1.01‐1.09)
Hypertension	27.2	42.3	1.45 (1.28‐1.64)	1.36 (1.20‐1.54)
Diabetes	11.3	18.2		1.27 (1.09‐1.48)
Obesity	17.9	21.9		1.31 (1.12‐1.52)
Smoking				
Never	21.3	19.4		1.00
≤20 pack‐year	33.2	26.8		1.04 (0.87‐1.24)
>20‐40 pack‐year	26.6	29.2		1.12 (0.95‐1.33)
>40 pack‐year	18.9	24.6		1.09 (0.91‐1.31)
Daily alcohol intakes				
Never	18.3	17.9		1.00
≤15 g	9.1	8.5		0.98 (0.76‐1.27)
>15‐30 g	31.5	33.8		1.05 (0.87‐1.26)
>30 g	41.1	39.8		1.03 (0.85‐1.25)
Women				
Total number	21 063	279		
Occupational class				
Blue‐collar industry				
Blue‐collar worker	21.8	20.8	1.00	1.00
Service worker	8.4	8.2	1.20 (0.73‐1.96)	1.21 (0.74‐1.99)
Professional	0.5	0.4	1.06 (0.14‐7.78)	1.10 (0.15‐8.04)
Managers	0.4	0.4	0.83 (0.11‐6.03)	0.83 (0.11‐6.04)
Service industry				
Blue‐collar worker	5.1	10.4	2.29 (1.45‐3.60)	2.32 (1.48‐3.66)
Service worker	30.3	31.2	1.24 (0.88‐1.74)	1.29 (0.91‐1.81)
Professional	0.8	1.4	2.25 (0.80‐6.31)	2.31 (0.82‐6.48)
Managers	0.4	1.1	2.51 (0.77‐8.16)	2.73 (0.84‐8.91)
White‐collar industry				
Blue‐collar worker	0.9	0.4	0.49 (0.07‐3.57)	0.49 (0.07‐3.55)
Service worker	14.5	14.7	1.32 (0.87‐1.99)	1.33 (0.88‐2.02)
Professional	16.8	11.1	0.90 (0.57‐1.41)	0.90 (0.58‐1.42)
Manager			NA	NA
Age, mean (SD), y	58 (16)	62 (12)	1.02 (1.01‐1.03)	1.01 (1.00‐1.02)
Year of admission, mean (SD)	2010 (3)	2010 (3)	1.01 (0.94‐1.09)	1.02 (0.95‐1.10)
Hypertension	26.4	34.9	1.22 (0.94‐1.60)	1.16 (0.89‐1.52)
Diabetes	7.2	11.0		1.31 (0.88‐1.95)
Obesity	16.0	19.4		1.19 (0.87‐1.64)
Smoking				
Never	73.7	82.1		1.00
≤20 pack‐year	19.0	9.7		0.58 (0.38‐0.89)
>20‐40 pack‐year	6.0	7.2		1.18 (0.73‐1.91)
>40 pack‐year	1.4	1.1		0.69 (0.22‐2.20)
Daily alcohol intakes				
Never	57.2	67.1		1.00
≤15 g	15.6	12.6		0.86 (0.56‐1.34)
>15‐30 g	19.9	15.1		0.81 (0.54‐1.22)
>30 g	7.3	5.2		0.81 (0.44‐1.47)

Abbreviation: NA, not available.

a Data were estimated with 5 imputed datasets with study subjects after 2005 owing to the data limitation for lifestyle‐related disease (hypertension, diabetes, and obesity). The percentage may not total 100 because of rounding and multiple imputation.

b Unconditional logistic regression with multiple imputation, adjusted for age and year of admission (confounders) and hypertension (mediators, model 4).

c Additional adjustment for diabetes, obesity, smoking, and alcohol consumption (mediators, model 5).

**Figure 4:**
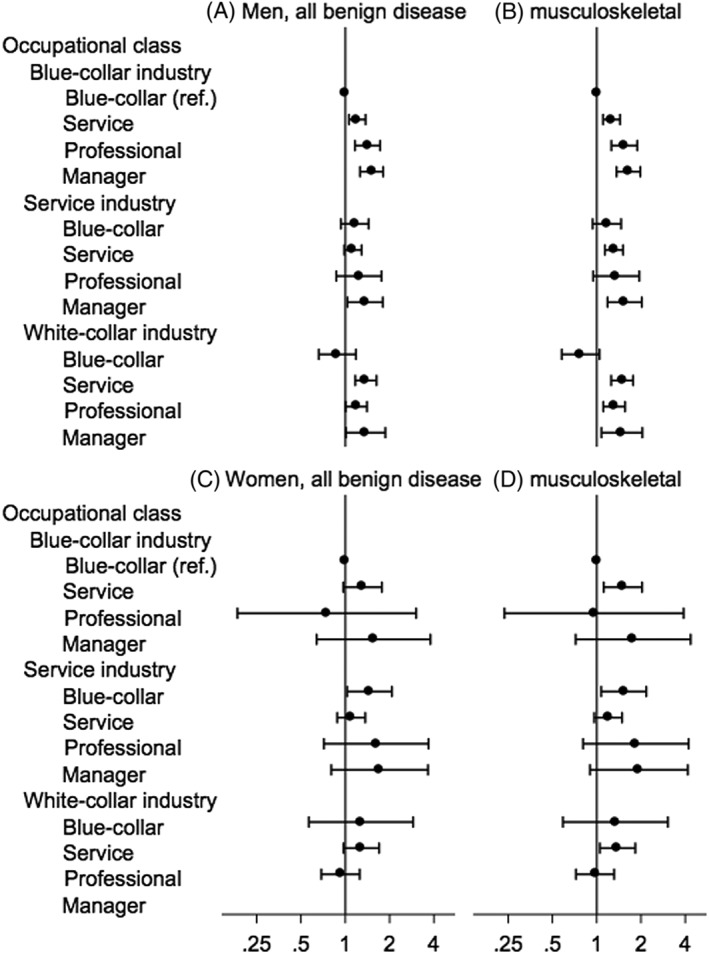
Sensitivity analysis with alternative control groups. The odds ratio (dot) and 95% confidence interval (bar) were estimated by unconditional logistic regression, adjusted for age and year of admission with 5 imputed data. Male and female control groups were, respectively, (A, C) patients diagnosed with all benign diseases and (B, D) patients diagnosed with musculoskeletal disease. The numbers of cases and controls were, respectively, as follows: (A) 2703 and 852 997 for men and (C) 613 and 445 210 for women (all benign disease controls); (B) 2703 and 99 317 for men and (D) 613 and 50 893 for women (musculoskeletal disease controls)

In sensitivity analyses, although the precise ORs and 95% CIs differed according to the analytic model and study population, the directions of the association (ie, higher risk with higher occupational class) were identical (Figure 4 and Table S2). The result with complete data also showed the same pattern (Figure S1). The correlation between hypertension, diabetes, and obesity were all significant (pairwise correlation; all *P* values < .001). The profile of patients treated in Rosai hospitals appeared to be nationally representative (Table S3). The average length of longest held jobs was over 20 years (Table S4).

## 4 DISCUSSION

We found an elevated risk of renal cell cancer among high status occupations (managers and professionals) in men across all industry categories, suggesting that high job stress may partially be associated with the risk of renal cell cancer. We also found, for the first time, that hypertension is a relevant independent risk factor for renal cell cancer in Japan. Furthermore, the risk for renal cell cancer associated with higher occupational class was potentially mediated through the risk for renal cell cancer associated with stress‐related risk factors—viz, hypertension as well as diabetes and obesity. A similar tendency was found for women working in the service and sales industry, although the effects were marginal.

Job stress may be related to risk of renal cell cancer through both direct and indirect causal pathways. The direct pathway posits that job stress increases risk through direct biological or mechanical stimulus to cancer stem cells (eg, oxidative stress).^41^, ^42^ Although the association between occupation and renal cell cancer was substantially explained by hypertension and other potential mediators (diabetes and obesity), some significant associations in blue‐collar and white‐collar industries persisted among men in the present study. This residual association suggests that the direct pathway may be partially pertinent for renal cell cancer.

The indirect pathway posits that job stress may increase the risk of renal cell cancer via risk factors potentially influenced by stressful occupations, eg, cigarette smoking or the prevalence of hypertension. In fact, previous studies have suggested that psychological factors (eg, chronic or work environmental stress) can increase such lifestyle‐related diseases.^43^, ^44^, ^45^, ^46^ In the present study, the prevalence of those who smoked more than 40 pack‐years was higher in the managers than nonmanagers (25% versus 11%), and the prevalence of hypertension was greater in the managers (37% versus 27%).

In Japanese society, the concept of “hospitality” or *omotenashi* is emphasized in the service industry. Because of these expectations, those in managerial positions (or in the position of supervising other workers) may be particularly vulnerable to stress stemming from striving to meet customer expectations. In some instances, this situation has even led to death from overwork, referred to as *karoshi*. Such stress has been found to affect work‐life balance among high occupational class workers.^47^ By contrast, Whitehall studies showed that poorer health (eg, cardiovascular disease) is associated with low control at work,^48^ which is usually the case for blue‐collar workers in western contexts. Low control at work was also associated with less leisure‐time physical activity.^49^ Although our study is one of the largest case‐control studies of renal cell cancer reported in Japan (3316 cases) and the profile of patients treated in Rosai hospitals appeared to be nationally representative^50^ (Table S3), it represents less than 1% of the total incidence in the country as a whole. Hence, the generalizability of our findings to the rest of the country may be limited.

The strengths of our study include the large sample size and the detailed job information that enabled us to create occupational classes into meaningful categories by both industrial and occupational standard classifications. Another strength is the low job turn over in Japan, ie, the percentage of workers changing jobs is lower compared with other countries. In fact, prior data show that an average of 50% of men and 30% of women at their working age did not change their first job, and 20% of men and 20% of women changed only once during the age^51^ from 15 to 64. Our occupational information consisted of current and up to 3 former jobs, and we chose the longest career as a proxy of job stress (the average length of longest held jobs was over 20 y; Table S4); therefore, in the sense of lifelong stress, our captured stress would be more relevant than stress measured at baseline only once in cohort studies.^21^ In fact, a case‐control study from Canada also found a significant association between job stress and cancer incidence at other sites.^52^ Furthermore, a stressful working environment of the high occupational classes in Japan also enabled us to detect the association between higher occupational class, possibly linked to job stress, and the incidence of renal cell cancer.^22^

There are some limitations in our study. First, in any hospital‐based case‐control study, the selection of hospital controls may introduce selection bias in either direction (ie, toward or away from the null). However, sensitivity analysis, including controls diagnosed with all benign diseases (except malignant neoplasms) or only controls diagnosed with musculoskeletal disease, resulted in the same direction to increase the risk. Additionally, one‐third of missing data may have introduced selection bias in either direction—even though the missing information were multiply imputed; however, the sensitivity analysis with complete data showed the same pattern. There might also be a potential recall bias in the self‐reported information at the time of admission (eg, occupational history). However, the association of job stress and renal cell cancer was not widely known at that time. In addition, the questionnaires did not ask patients to report job stress, and the study subjects did not know the aim of our study. Therefore, the recall bias for occupational history may not be at play between the cases and controls, and this limitation might not affect our conclusion.

Second, occupational class is not a perfect proxy for job stress, and we could not directly assess job stress because our hospital electronic medical record data did not include an assessment of stress. Higher occupational class may also reflect anxiety, depression, and other mental health conditions.^29^ Kawakami et al also speculated that job commitment in these high positions might decrease the opportunities for investing in healthier behaviors such as leisure‐time physical activity.^26^ Physical activity has been found to be a protective factor for the risk of renal cell cancer.^53^ A previous study found that the pattern of leisure‐time physical activity differs in Japan compared with western contexts, viz, the highest levels of exercise were reported by clerical workers, while the lowest levels were reported among managerial workers and blue‐collar workers.^54^ In the same study, the highest levels of weekly physical activity, including occupational physical activity, were reported by blue‐collar workers and the lowest levels among professional and managerial workers.^54^ These findings suggest that higher occupational class may be associated with sedentary lifestyle behaviors, and that sedentary lifestyle may increase the risk of renal cell cancer. However, we could not assess potential mediation by physical activity/sedentary behavior because of the limitation of our dataset. Therefore, future studies should investigate the accumulation of stress on renal cell cancer, incorporating other aspects of job stress and the intervention on mental health, as well as possible residual confounding factors including physical activity, genetic, and nutrition factors, as well as dehydration.^26^, ^54^, ^55^, ^56^

In summary, higher occupational class, which might be linked to job stress, was associated with increased odds for renal cell cancer, particularly among men, via mediation by lifestyle‐related factors such as hypertension. Stress management interventions in the workplace might be a possible approach to complement existing lifestyle interventions aimed at reducing the risk of renal cell cancer.

## FUNDING

This study is supported by Industrial Disease Clinical Research Grants from Ministry of Health, Labour, and Welfare (No. 170201‐01). C.T.F. received a postdoctoral fellowship from the Fonds de Recherche du Quebec—Sante. A.G.C. was supported by the National Institutes of Health (NIH) 3R25CA057711. Its contents are solely the responsibility of the authors and do not necessarily represent the official views of the NIH.

## CONFLICTS OF INTEREST

The authors declare no conflicts of interest.

## ETHICS APPROVAL AND INFORMED CONSENT

Written informed consent was obtained before patients completed the questionnaires. The Research Ethics Committees of Graduate School of Medicine, The University of Tokyo, Tokyo (Protocol Number 3890‐3) and Kanto Rosai Hospital, Kanagawa, Japan (Protocol Number 2014‐38) approved the study.

## DISCLAIMER

None.

## AUTHOR CONTRIBUTIONS

Conceptualization: Masayoshi Zaitsu, Ichiro Kawachi

Formal analysis: Masayoshi Zaitsu

Writing—review and editing: Masayoshi Zaitsu, Adolfo G. Cuevas, Claudia Trudel‐Fitzgerald, Takumi Takeuchi, Yasuki Kobayashi, Ichiro Kawachi

Writing—original draft: Masayoshi Zaitsu

## Supporting information


**Figure S1.** **Risk for each occupational class associated with renal cell cancer with complete data.** The control group comprised patients diagnosed with musculoskeletal disease (90.7%) and skin diseases (9.3%). The odds ratio (dot) and 95% confidence interval (bar) were estimated by unconditional logistic regression adjusted for age and year of admission (model 1). Among men, even after controlling for smoking and alcohol consumption, the elevated odds with higher occupational class (professionals and managers) remained significantly associated with the risk for renal cell cancer across all industries (model 2). Among females, similar pattern was observed, particularly in service industries. OR, odds ratio; CI, confidence interval.
**Table S1.** **Characteristics of patients with complete and incomplete data.**
**Table S2.** Odds ratios in each occupational class associated with risk for renal cell cancer estimated with different control groups.
**Table S3.** The distribution of occupational class and industrial cluster among all patients treated in the Rosai hospital group between 2009 and 2016 compared to the national statistics.
**Table S4.** Average lengths of longest held jobs.

## References

[1] Washio M , Mori M , Mikami K , et al. Cigarette smoking and other risk factors for kidney cancer death in a Japanese population: Japan collaborative cohort study for evaluation of cancer risk (JACC study). Asian Pac J Cancer Prev. 2013;14(11):6523‐6528. 10.7314/apjcp.2013.14.11.6523 24377561

[2] Washio M , Mori M , Khan M , et al. Diabetes mellitus and kidney cancer risk: the results of Japan collaborative cohort study for evaluation of cancer risk (JACC study). Int J Urol. 2007;14(5):393‐397. 17511719 10.1111/j.1442-2042.2007.01744.x

[3] Znaor A , Lortet‐Tieulent J , Laversanne M , Jemal A , Bray F . International variations and trends in renal cell carcinoma incidence and mortality. Eur Urol. 2015;67(3):519‐530. 25449206 10.1016/j.eururo.2014.10.002

[4] Cancer Registry and Statistics . Cancer Information Service, National Cancer Center, Japan. 2015.

[5] Heikkilä K , Nyberg ST , Fransson EI , et al. Job strain and tobacco smoking: an individual‐participant data meta‐analysis of 166,130 adults in 15 European studies. PLoS one. 2012;7(7):e35463. 22792154 10.1371/journal.pone.0035463 PMC3391192

[6] Liu MY , Li N , Li WA , Khan H . Association between psychosocial stress and hypertension: a systematic review and meta‐analysis. Neurol Res. 2017;39(6):573‐580. 28415916 10.1080/01616412.2017.1317904

[7] Jääskeläinen A , Kaila‐Kangas L , Leino‐Arjas P , et al. Psychosocial factors at work and obesity among young Finnish adults: a cohort study. J Occup Environ Med. 2015;57(5):485‐492. 25793463 10.1097/JOM.0000000000000432

[8] Chow W , Gridley G , Fraumeni J , Jarvholm B . Obesity, hypertension, and the risk of kidney cancer in men. N Engl J Med. 2000;343(18):1305‐1311. 11058675 10.1056/NEJM200011023431804

[9] Chow WH , Dong LM , Devesa SS . Epidemiology and risk factors for kidney cancer. Nat Rev Urol. 2010;7(5):245‐257. 20448658 10.1038/nrurol.2010.46 PMC3012455

[10] Pan SY , DesMeules M , Morrison H , Wen SW , Canadian Canc Registries Epidemiol . Obesity, high energy intake, lack of physical activity, and the risk of kidney cancer. Cancer Epidemiol Biomarkers Prev. 2006;15(12):2453‐2460. 17164370 10.1158/1055-9965.EPI-06-0616

[11] Sanfilippo KM , McTigue KM , Fidler CJ , et al. Hypertension and obesity and the risk of kidney cancer in 2 large cohorts of US men and women. Hypertension. 2014;63(5):934‐941. 24637660 10.1161/HYPERTENSIONAHA.113.02953 PMC4098147

[12] Sawada N , Inoue M , Sasazuki S , et al. Body mass index and subsequent risk of kidney cancer: a prospective cohort study in Japan. Ann Epidemiol. 2010;20(6):466‐472. 20470974 10.1016/j.annepidem.2010.03.008

[13] Sun LM , Kuo HT , Jeng LB , Lin CL , Liang JA , Kao CH . Hypertension and subsequent genitourinary and gynecologic cancers risk: a population‐based cohort study. Medicine (Baltimore). 2015;94(16):e753. 25906108 10.1097/MD.0000000000000753 PMC4602691

[14] Song M , Giovannucci E . Preventable incidence and mortality of carcinoma associated with lifestyle factors among white adults in the United States. JAMA Oncol. 2016;2(9):1154‐1161. 27196525 10.1001/jamaoncol.2016.0843 PMC5016199

[15] Lutgendorf SK , Sood AK , Antoni MH . Host factors and cancer progression: biobehavioral signaling pathways and interventions. J Clin Oncol. 2010;28(26):4094‐4099. 20644093 10.1200/JCO.2009.26.9357 PMC2940426

[16] Thaker PH , Lutgendorf SK , Sood AK . The neuroendocrine impact of chronic stress on cancer. Cell Cycle. 2007;6(4):430‐433. 17312398 10.4161/cc.6.4.3829

[17] Trudel‐Fitzgerald C , Chen Y , Singh A , Okereke OI , Kubzansky LD . Psychiatric, psychological, and social determinants of health in the nurses' health study cohorts. Am J Public Health. 2016;106(9):1644‐1649. 27459447 10.2105/AJPH.2016.303318 PMC4981800

[18] Kubzansky LD , Winning A , Kawachi I . In: Berkman LF, Kawachi I, Glymour MM, eds. Affective States and Health. New York: Social Epidemiology, Oxford University Press; 2014:320‐364.

[19] Schernhammer ES , Hankinson SE , Rosner B , et al. Job stress and breast cancer risk: the nurses' health study. Am J Epidemiol. 2004;160(11):1079‐1086. 15561987 10.1093/aje/kwh327

[20] Trudel‐Fitzgerald C , Poole EP , Idahl A , et al. The association of work characteristics with ovarian cancer risk and mortality. Psychosom Med. 2017;79(9):1059‐1067. 28306624 10.1097/PSY.0000000000000464 PMC5601015

[21] Heikkilä K , Nyberg ST , Theorell T , et al. Work stress and risk of cancer: meta‐analysis of 5700 incident cancer events in 116,000 European men and women. BMJ. 2013;346(feb07 1):f165. 23393080 10.1136/bmj.f165 PMC3567204

[22] Suzuki E , Kashima S , Kawachi I , Subramanian SV . Social and geographical inequalities in suicide in Japan from 1975 through 2005: a census‐based longitudinal analysis. PLoS one. 2013;8(5):e63443. 23671679 10.1371/journal.pone.0063443 PMC3646025

[23] Wada K , Kondo N , Gilmour S , et al. Trends in cause specific mortality across occupations in Japanese men of working age during period of economic stagnation, 1980‐2005: retrospective cohort study. BMJ. 2012;344(mar06 3):e1191. 22396155 10.1136/bmj.e1191 PMC3295860

[24] Eguchi H , Wada K , Prieto‐Merino D , Smith DR . Lung, gastric and colorectal cancer mortality by occupation and industry among working‐aged men in Japan. Sci Rep. 2017;7:43204. 28230191 10.1038/srep43204 PMC5322319

[25] Tanaka H , Toyokawa S , Tamiya N , Takahashi H , Noguchi H , Kobayashi Y . Changes in mortality inequalities across occupations in Japan: a national register based study of absolute and relative measures, 1980‐2010. BMJ Open. 2017;7(9). e015764‐2016‐015764 10.1136/bmjopen-2016-015764 PMC5588999 28877942

[26] Kawakami N , Haratani T , Kobayashi F , et al. Occupational class and exposure to job stressors among employed men and women in Japan. J Epidemiol. 2004;14(6):204‐211. 15617394 10.2188/jea.14.204 PMC8784243

[27] Inoue A , Kawakami N , Tsuchiya M , Sakurai K , Hashimoto H . Association of occupation, employment contract, and company size with mental health in a national representative sample of employees in Japan. J Occup Health. 2010;52(4):227‐240. 20526043 10.1539/joh.o10002

[28] Tsutsumi A , Kayaba K , Tsutsumi K , Igarashi M , Jichi medical school cohort study group . Association between job strain and prevalence of hypertension: a cross sectional analysis in a Japanese working population with a wide range of occupations: the Jichi Medical School cohort study. Occup Environ Med. 2001;58(6):367‐373. 11351051 10.1136/oem.58.6.367 PMC1740148

[29] Otsuka K , Kato S . Relationship between diagnostic subtypes of depression and occupation in Japan. Psychopathology. 2000;33(6):324‐328. 11060517 10.1159/000029166

[30] Johnson JV , Stewart WF . Measuring work organization exposure over the life course with a job‐exposure matrix. Scand J Work Environ Health. 1993;19(1):21‐28. 10.5271/sjweh.1508 8465168

[31] Mannetje A , Kromhout H . The use of occupation and industry classifications in general population studies. Int J Epidemiol. 2003;32(3):419‐428. 12777430 10.1093/ije/dyg080

[32] Zaitsu M , Nakamura F , Toyokawa S , et al. Risk of alcohol consumption in bladder cancer: case‐control study from a nationwide inpatient database in Japan. Tohoku J Exp Med. 2016;239(1):9‐15. 27098227 10.1620/tjem.239.9

[33] Zaitsu M , Kawachi I , Takeuchi T , Kobayashi Y . Alcohol consumption and risk of upper‐tract urothelial cancer. Cancer Epidemiol. 2017;48:36‐40. 28364670 10.1016/j.canep.2017.03.002

[34] Prakash KC , Neupane S , Leino‐Arjas P , et al. Work‐related biomechanical exposure and job strain as separate and joint predictors of musculoskeletal diseases: a 28‐year prospective follow‐up study. Am J Epidemiol. 2017;186(11):1256‐1267. 29206989 10.1093/aje/kwx189

[35] Galobardes B , Shaw M , Lawlor DA , Lynch JW , Davey Smith G . Indicators of socioeconomic position (part 2). J Epidemiol Community Health. 2006;60(2):95‐101. 16415256 10.1136/jech.2004.028092 PMC2566160

[36] Martikainen P , Lahelma E , Marmot M , Sekine M , Nishi N , Kagamimori S . A comparison of socioeconomic differences in physical functioning and perceived health among male and female employees in Britain, Finland and Japan. Soc Sci Med. 2004;59(6):1287‐1295. 15210099 10.1016/j.socscimed.2004.01.005

[37] Jackson CL , Redline S , Kawachi I , Williams MA , Hu FB . Racial disparities in short sleep duration by occupation and industry. Am J Epidemiol. 2013;178(9):1442‐1451. 24018914 10.1093/aje/kwt159 PMC3888251

[38] Hayati Rezvan P , Lee KJ , Simpson JA . The rise of multiple imputation: a review of the reporting and implementation of the method in medical research. BMC Med Res Methodol. 2015;15 30–015–0022‐1. 10.1186/s12874-015-0022-1 PMC4396150 25880850

[39] Royston P . Multiple imputation of missing values: further update of ice, with an emphasis on categorical variables. Stata Journal. 2009;9(3):466‐477.

[40] Zaitsu M , Kawachi I , Ashida T , Kondo K , Kondo N . Participation in community group activities among older adults: is diversity of group membership associated with better self‐rated health? J Epidemiol. 2018 (published online Apr 28, 2018). 10.2188/jea.JE20170152 PMC6192976 29709889

[41] Hori Y , Oda Y , Kiyoshima K , et al. Oxidative stress and DNA hypermethylation status in renal cell carcinoma arising in patients on dialysis. J Pathol. 2007;212(2):218‐226. 17451187 10.1002/path.2176

[42] Ganesamoni R , Bhattacharyya S , Kumar S , et al. Status of oxidative stress in patients with renal cell carcinoma. J Urol. 2012;187(4):1172‐1176. 22335872 10.1016/j.juro.2011.11.105

[43] Cuevas AG , Williams DR , Albert MA . Psychosocial factors and hypertension. A Review of the Literature Cardiol Clin. 2017;35(2):223‐230. 28411896 10.1016/j.ccl.2016.12.004 PMC5407387

[44] Chandola T , Brunner E , Marmot M . Chronic stress at work and the metabolic syndrome: prospective study. BMJ. 2006;332(7540):521‐525. 16428252 10.1136/bmj.38693.435301.80 PMC1388129

[45] Bergmann N , Gyntelberg F , Faber J . The appraisal of chronic stress and the development of the metabolic syndrome: a systematic review of prospective cohort studies. Endocr Connect. 2014;3(2):R55‐R80. 24743684 10.1530/EC-14-0031 PMC4025474

[46] Trudel‐Fitzgerald C , Gilsanz P , Mittleman MA , Kubzansky LD . Dysregulated blood pressure: can regulating emotions help? Curr Hypertens Rep. 2015;17(12):92 10.1007/s11906-015-0605-6 26520446 PMC4764066

[47] Eguchi H , Wada K , Smith DR . Recognition, compensation, and prevention of karoshi, or death due to overwork. J Occup Environ Med. 2016;58(8):e313‐e314. 27500999 10.1097/JOM.0000000000000797

[48] Bosma H , Peter R , Siegrist J , Marmot M . Two alternative job stress models and the risk of coronary heart disease. Am J Public Health. 1998;88(1):68‐74. 9584036 10.2105/ajph.88.1.68 PMC1508386

[49] Gimeno D , Elovainio M , Jokela M , De Vogli R , Marmot MG , Kivimaki M . Association between passive jobs and low levels of leisure‐time physical activity: the Whitehall II cohort study. Occup Environ Med. 2009;66(11):772‐776. 10.1136/oem.2008.045104 19528047 PMC3226945

[50] Statistics Bureau Ministry of Internal Affairs and Communications . Labour Force Survey. Retrieved from http://www.stat.go.jp/english/data/roudou/qa.htm. Accessed December 13, 2017.

[51] White paper on the labour economy. 2014 Retrieved from http://www.mhlw.go.jp/english/wp/l-economy/2014/dl/2014outline.pdf. Accessed April 5, 2018.

[52] Blanc‐Lapierre A , Rousseau MC , Weiss D , El‐Zein M , Siemiatycki J , Parent ME . Lifetime report of perceived stress at work and cancer among men: a case‐control study in Montreal, Canada. Prev Med. 2016;96:28‐35. 27923666 10.1016/j.ypmed.2016.12.004

[53] Moore SC , Chow WH , Schatzkin A , et al. Physical activity during adulthood and adolescence in relation to renal cell cancer. Am J Epidemiol. 2008;168(2):149‐157. 18468990 10.1093/aje/kwn102 PMC2878095

[54] Takao S , Kawakami N , Ohtsu T , Japan work stress and health cohort study group . Occupational class and physical activity among Japanese employees. Soc Sci Med. 2003;57(12):2281‐2289. 14572837 10.1016/s0277-9536(03)00134-5

[55] Vogelzang NJ , Stadler WM . Kidney cancer. Lancet. 1998;352(9141):1691‐1696. 9853456 10.1016/S0140-6736(98)01041-1

[56] Washio M , Mori M , Sakauchi F , et al. Risk factors for kidney cancer in a Japanese population: findings from the JACC study. J Epidemiol. 2005;15(Suppl 2):S203‐S211. 16127235 10.2188/jea.15.S203 PMC8639037

