# Supplementary material for: Occupational class and risk of renal cell cancer
Source: Health Sci Rep. 2018 May 16;1(6):e49. doi: 10.1002/hsr2.49 (PMC6266576; doi:10.1002/hsr2.49)
Supplement: Supplementary file 1 — Figure S1. Risk for each occupational class associated with renal cell cancer with complete data. The control group comprised patients diagnosed with musculoskeletal disease (90.7%) and skin diseases (9.3%). The odds ratio (dot) and 95% confidence interval (bar) were estimated by unconditional logistic regression adjusted for age and year of admission (model 1). Among men, even after controlling for smoking and alcohol consumption, the elevated odds with higher occupational class (professionals and managers) remained significantly associated with the risk for renal cell cancer across all industries (model 2). Among females, similar pattern was observed, particularly in service industries. OR, odds ratio; CI, confidence interval. Table S1. Characteristics of patients with complete and incomplete data. Table S2. Odds ratios in each occupational class associated with risk for renal cell cancer estimated with different control groups. Table S3. The distribution of occupational class and industrial cluster among all patients treated in the Rosai hospital group between 2009 and 2016 compared to the national statistics. Table S4. Average lengths of longest held jobs. [file HSR2-1-e49-s001.docx]

**Figure S1. Risk for each occupational class associated with renal cell cancer with complete data.** The control group comprised patients diagnosed with musculoskeletal disease (90.7%) and skin diseases (9.3%). The odds ratio (dot) and 95% confidence interval (bar) were estimated by unconditional logistic regression adjusted for age and year of admission (model 1). Among men, even after controlling for smoking and alcohol consumption, the elevated odds with higher occupational class (professionals and managers) remained significantly associated with the risk for renal cell cancer across all industries (model 2). Among females, similar pattern was observed, particularly in service industries. OR, odds ratio; CI, confidence interval.


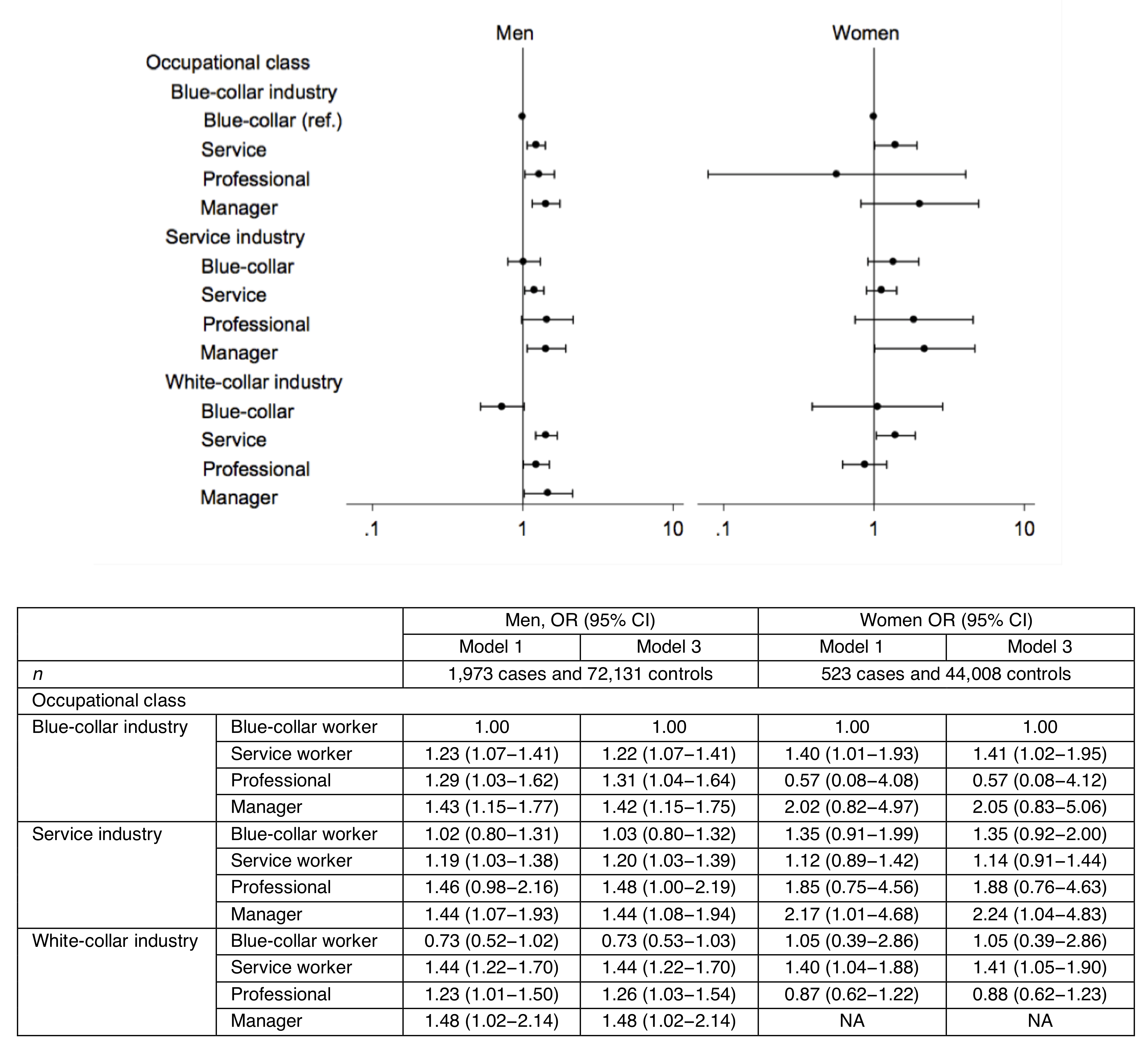


**Table S1. Characteristics of patients with complete and incomplete data**

| Characteristics | Data not completed† | Completed† | P-Value‡ |
| --- | --- | --- | --- |
| **Overall (2005-2016)** |  |  |  |
| Total number | *n* = 53,099 | *n* = 118,635 |  |
| Case | 820 (1.5) | 2,496 (2.1) | < .001 |
| Female | 12,575 (24) | 44,531 (38) | < .001 |
| Age, mean (SD) | 45 (16) | 55 (16) | < .001 |
|  |  |  |  |
| Occupational class | n=32,940 | n=118,635 |  |
| Blue-collar industry |  |  |  |
| Blue-collar workers | 9,716 (29) | 44,194 (37) | < .001 |
| Service workers | 3,749 (11) | 14,119 (12) |  |
| Professionals | 1,129 (3.4) | 3,270 (2.3) |  |
| Managers | 745 (2.3) | 2,695 (2.3) |  |
| Service industry |  |  |  |
| Blue-collar workers | 1,521 (4.6) | 5,470 (4.6) |  |
| Service workers | 6,002 (18) | 22,253 (19) |  |
| Professionals | 380 (1.2) | 1,063 (0.9) |  |
| Managers | 403 (1.2) | 1,453 (1.2) |  |
| White-collar industry |  |  |  |
| Blue-collar workers | 1,111 (3.4) | 2,869 (2.4) |  |
| Service workers | 3,849 (12) | 10,570 (8.9) |  |
| Professionals | 4,025 (12) | 9,991 (8.4) |  |
| Managers | 310 (0.9) | 684 (0.6) |  |
|  |  |  |  |
| Smoking | n=29,407 | n=118,635 |  |
| Never | 14,854 (51) | 55,189 (47) | < .001 |
| ≤ 20 pack-years | 9,355 (32) | 26,183 (22) |  |
| > 20 to 40 pack-years | 3,586 (12) | 22,476 (19) |  |
| > 40 pack-years | 1,612 (4.3) | 14,787 (12) |  |
|  |  |  |  |
| Daily alcohol intakes | n=4,491 | n=118,635 |  |
| Never | 2,521 (56) | 54,257 (46) | < .001 |
| ≤ 15 g | 4 (0.1) | 3,012 (2.5) |  |
| > 15 to 30 g | 919 (20) | 32,035 (27) |  |
| > 30 g | 1,047 (23) | 29,331 (25) |  |
|  |  |  |  |
| **Subgroup (2005-2016)** |  |  |  |
| Hypertension | *n* = 11,580 | *n* = 42,008 |  |
| Yes | 1,879 (16) | 13,562 (32) | < .001 |
| Diabetes | n=11,579 | n=42,008 |  |
| Yes | 762 (6.6) | 4,755 (11) | < .001 |
| Obesity | n=11,577 | n=42,008 |  |
| Yes | 2,509 (22) | 7,238 (17) | < .001 |

† The percentage may not total 100 because of rounding. The number used for each background characteristics may not equal to the total number because of data missing. The study period from April 1984 to March 2016 was divided into 2-year financial years.

‡ P-values are for t-test and chi-squared test.

**Table S2. Odds ratios in each occupational class associated with risk for renal cell cancer estimated with different control groups**

|  | Controls with all benign diseases† | | Controls with musculoskeletal disease | |
| --- | --- | --- | --- | --- |
| Characteristics | Model 1‡ | Model 3§ | Model 1‡ | Model 3§ |
| **Men** | 2,703 cases and 852,997 controls | | 2,703 cases and 99,317 controls | |
| Occupational class |  |  |  |  |
| Blue-collar industry |  |  |  |  |
| Blue-collar worker | 1.00 | 1.00 | 1.00 | 1.00 |
| Service worker | 1.21 (1.06−1.37) | 1.20 (1.05−1.37) | 1.26 (1.10−1.44) | 1.26 (1.10−1.44) |
| Professional | 1.42 (1.16−1.72) | 1.41 (1.16−1.72) | 1.54 (1.26−1.89) | 1.55 (1.27−1.90) |
| Manager | 1.51 (1.26−1.81) | 1.50 (1.25−1.80) | 1.64 (1.36−1.98) | 1.63 (1.36−1.97) |
| Service industry |  |  |  |  |
| Blue-collar worker | 1.16 (0.93−1.44) | 1.17 (0.94−1.45) | 1.17 (0.94−1.46) | 1.18 (0.94−1.47) |
| Service worker | 1.13 (0.98−1.29) | 1.13 (0.99−1.29) | 1.31 (1.14−1.51) | 1.31 (1.14−1.51) |
| Professional | 1.24 (0.87−1.76) | 1.25 (0.88−1.78) | 1.36 (0.95−1.95) | 1.38 (0.96−1.98) |
| Manager | 1.36 (1.03−1.80) | 1.36 (1.03−1.80) | 1.55 (1.18−2.03) | 1.55 (1.19−2.03) |
| White-collar industry |  |  |  |  |
| Blue-collar worker | 0.88 (0.66−1.18) | 0.88 (0.66−1.18) | 0.78 (0.58−1.04) | 0.78 (0.58−1.05) |
| Service worker | 1.38 (1.17−1.63) | 1.38 (1.17−1.63) | 1.49 (1.25−1.77) | 1.49 (1.26−1.77) |
| Professional | 1.19 (1.00−1.40) | 1.20 (1.02−1.42) | 1.31 (1.11−1.56) | 1.34 (1.13−1.59) |
| Manager | 1.38 (1.01−1.87) | 1.38 (1.02−1.88) | 1.48 (1.08−2.04) | 1.48 (1.08−2.05) |
|  |  |  |  |  |
| **Women** | 613 cases and 445,210 controls | | 613 cases and 50,893 controls | |
| Occupational class |  |  |  |  |
| Blue-collar industry |  |  |  |  |
| Blue-collar worker | 1.00 | 1.00 | 1.00 | 1.00 |
| Service worker | 1.31 (0.97−1.77) | 1.32 (0.97−1.78) | 1.51 (1.12−2.03) | 1.52 (1.12−2.05) |
| Professional | 0.75 (0.18−3.03) | 0.75 (0.19−3.04) | 0.96 (0.24−3.90) | 0.97 (0.24−3.95) |
| Manager | 1.55 (0.64−3.79) | 1.59 (0.65−3.88) | 1.77 (0.72−4.35) | 1.81 (0.74−4.47) |
| Service industry |  |  |  |  |
| Blue-collar worker | 1.46 (1.03−2.07) | 1.48 (1.04−2.10) | 1.53 (1.07−2.17) | 1.54 (1.08−2.20) |
| Service worker | 1.10 (0.88−1.37) | 1.12 (0.90−1.40) | 1.20 (0.96−1.49) | 1.22 (0.98−1.52) |
| Professional | 1.62 (0.72−3.68) | 1.64 (0.72−3.73) | 1.84 (0.81−4.21) | 1.87 (0.82−4.28) |
| Manager | 1.71 (0.80−3.65) | 1.77 (0.83−3.78) | 1.94 (0.90−4.16) | 2.01 (0.93−4.32) |
| White-collar industry |  |  |  |  |
| Blue-collar worker | 1.28 (0.56−2.89) | 1.29 (0.57−2.92) | 1.34 (0.59−3.04) | 1.34 (0.59−3.06) |
| Service worker | 1.28 (0.97−1.70) | 1.29 (0.98−1.71) | 1.39 (1.05−1.83) | 1.40 (1.06−1.85) |
| Professional | 0.93 (0.69−1.25) | 0.93 (0.69−1.26) | 0.97 (0.72−1.31) | 0.98 (0.73−1.32) |
| Manager | NA | NA | NA | NA |

NA: not available.

† Benign neoplasm (7.0%), circulatory disease (12.9%), digestive disease (12.4%), endocrine disease (3.4%), eye and ear disease (6.3%), genitourinary system disease (6.0%), infectious disease (2.7%), injury (15.0%), mental disease (0.8%), musculoskeletal disease (11.6%), nerve system disease (2.9%), respiratory disease (6.8%), skin diseases (1.4%), ill-defined conditions (1.7%), or other diseases such as congenital malformations (9.2%).

‡ Unconditional logistic regression with multiple imputation, adjusted for age and year of admission (confounders, model 1). Data were estimated with five imputed datasets.

§ Additional adjustment for smoking and alcohol consumption (mediators, model 3).

**Table S3. Distribution of occupational class and industrial cluster among all patients treated in the Rosai hospital group between 2009 and 2016 compared to national statistics**

|  | Men |  |  | Women |  |
| --- | --- | --- | --- | --- | --- |
| Characteristics | National† | Rosai hospitals‡ |  | National† | Rosai hospitals‡ |
| Occupational class, % | *n* = 3,597 × 10^5^ | *n* = 179,733 |  | *n* = 2,673 × 10^5^ | *n* = 92,361 |
| Blue-collar workers | 40.1 | 41.6 |  | 20.7 | 21.8 |
| Service workers | 41.0 | 38.5 |  | 61.2 | 57.2 |
| Professionals | 15.1 | 14.1 |  | 17.5 | 20.3 |
| Managers | 3.8 | 5.8 |  | 0.7 | 0.8 |
|  |  |  |  |  |  |
| Industrial cluster, % | *n* = 3,143 × 10^5^ | *n* = 179,733 |  | *n* = 2,382 × 10^5^ | *n* = 92,361 |
| Blue-collar industry | 44.1 | 56.2 |  | 18.8 | 26.5 |
| Service industry | 29.3 | 22.9 |  | 41.0 | 37.3 |
| White-collar industry | 26.6 | 20.9 |  | 40.2 | 36.2 |

† Data extracted from the Labor Force Survey, Statistics Bureau, Ministry of Internal Affairs and Communication (2009–2016). Values are for the annual average per 10,000 population between 2009–2016.

‡ All patients treated in Rosai hospitals within the strata between 2009–2016. Data were estimated with five imputed datasets.

**Table S4. Average lengths of longest held jobs**

|  | Men |  | Women |
| --- | --- | --- | --- |
| Characteristics† | *n* = 114,628 |  | *n* = 57,106 |
| Overall, mean (SE), year | 23 (<0.1) |  | 20 (0.1) |
|  |  |  |  |
| Each occupational class, mean (SE), year |  |  |  |
| Blue-collar industry |  |  |  |
| Blue-collar workers | 25 (0.1) |  | 27 (0.1) |
| Service workers | 21 (0.1) |  | 16 (0.2) |
| Professionals | 21 (0.2) |  | 14 (0.8) |
| Managers | 28 (0.3) |  | 27 (0.9) |
| Service industry |  |  |  |
| Blue-collar workers | 19 (0.3) |  | 16 (0.3) |
| Service workers | 20 (0.1) |  | 18 (0.1) |
| Professionals | 21 (0.5) |  | 15 (0.7) |
| Managers | 26 (0.4) |  | 28 (0.9) |
| White-collar industry |  |  |  |
| Blue-collar workers | 20 (0.2) |  | 15 (0.5) |
| Service workers | 21 (0.2) |  | 15 (0.2) |
| Professionals | 20 (0.2) |  | 17 (0.1) |
| Managers | 25 (0.4) |  |  |

† The longest held job was selected from the occupational history for each patient. The occupational history consisted of current and up to three former jobs. Data were estimated with five imputed datasets.
